# Adjusted Indirect and Mixed Comparisons of Interventions for the Patient-Reported Outcomes Measures (PROMs) of Disabled Adults: A Systematic Review and Network Meta-Analysis

**DOI:** 10.3390/ijerph18052406

**Published:** 2021-03-01

**Authors:** Yining Xu, Xin Li, Zhihong Sun, Yang Song, Julien S. Baker, Yaodong Gu

**Affiliations:** 1 Faculty of Sports Science, Ningbo University, Ningbo 315211, China; xuyining_nbu@foxmail.com (Y.X.); andywutong@foxmail.com (Z.S.); 2 Research Academy of Grand Health, Ningbo University, Ningbo 315211, China; lixin_nbu@foxmail.com; 3 Faculty of Engineering, University of Szeged, 6724 Szeged, Hungary; yang.song@uni-obuda.hu; 4 Centre for Health and Exercise Science Research, Department of Sport, Physical Education and Health, Hong Kong Baptist University, Hong Kong, China

**Keywords:** people with disabilities, disabled, handicap, patient-reported outcome measures, network meta-analysis

## Abstract

This systematic review adopted the Preferred Reporting Items for Systematic Reviews and Meta-Analyses Statement (PRISMA) guidelines and used the method of network meta-analysis to compare the effects of different types of interventions from different perspectives which were abilities of daily life activity, psychological health, social functioning, and overall life quality. The eligibility criteria were: (1) Participants were adults above 18 years old with disabilities; (2) Interventions could be classified into active exercise, passive therapy, psychological education, psychosocial support program, multi-disciplinary program, and usual care; (3) Outcomes should be the patient-reported outcome measures (PROMs) that could be classified into abilities of daily life activity, psychological health, social functioning, and overall life quality; (4) Randomized designed and published in English. The keywords and their search field were: (1) “people with disabilities/disability”, “disabled”, “handicapped”, or “disable people” in titles or abstracts; (2) AND “randomized” or “randomised” in titles or abstracts; (3) NOT “design”, “protocol”, or “review” in titles. After searching in databases of Medline (EBSCO), PubMed, CINAHL, and Ovid, 16 studies were included. As a result, active exercise and passive therapy are most likely to be the best interventions for overall life quality, psychological education and passive therapy are most likely to be the best interventions for abilities of daily life activity, and psychosocial support programs are most likely to be the best intervention for psychological health and social functioning.

## 1. Introduction

Disability, whose definition is the inability to engage in complex or gainful activity due to physical or mental impairment, is a result of the interactions between the impaired individual and the environment in which they function. The determination of disability includes assessments of impairment, associated functional impairments, and any impact on the ability to perform activities of daily living and work. Evaluation of disability is an important aspect of social and clinical care. Accurate evaluation is significantly meaningful to the wellbeing of both patients and society, giving the impact of disability status on financial remuneration, return to work, personal and workplace productivity, and access to existing and future health care needs [1].

However, assessment of disability is complex, variable, and challenging even among clinicians experienced in disability determination. Many factors give rise to these challenges [2], the most important of which is that the determination of disability requires a synthesis of clinical and non-clinical information. Clinicians in practice have the unique opportunity to observe the effects of physical and mental impairments on their patients’ overall function, including the ability to work and live independently [3,4] and the first step in disability evaluation is the determination of medical impairment and its impact on the ability to perform activities of daily living, which can help in determining functional abilities and limitations. 

Being different from normal diseases, disabilities are usually irreversible. Therefore, to people with disabilities, the objective of treatments, therapies, education, and social care programs would be no longer to cure their disabilities than to help them regain their daily life quality. This systematic review and network meta-analysis pool the interventions belongs to active exercise, passive therapy, psychological education, psychosocial support program, and multi-disciplinary program together, comparing their effects from different perspectives which are the ability of daily life activities, psychological health, social functioning, and over life quality.

The population of this systematic review is determined to follow the definition of the term “disability” which follows the description given by the Social Security Administration (SSA) in 2017. According to the SSA, the definition of disability are as follows: (1) An alteration of an individual’s capacity to meet personal, social, or occupational demands or statutory or regulatory requirements because of an impairment [5]; (2) Activity limitations and/or participation restrictions in an individual with a health condition disorder or disease [6]; (3) The inability to engage in any substantial, gainful activity (SGA) because of a medically determinable physical or mental impairment(s), which can be expected to result in death or which has lasted or can be expected to last for a continuous period of not less than 12 months [3]; (4) A physical or mental impairment that substantially limits one or more major life activities; has a record of such an impairment; or is regarded as having such an impairment [7]; (5) A restriction or lack (resulting from an impairment) of ability to perform an activity in the manner or within the range considered normal for a human being. In conclusion, the SSA defines disability as “the inability to engage in any substantial, gainful activity because of a medically determinable physical or mental impairment(s), which could be expected to result in death or which has lasted or can be expected to last for a continuous period of not less than 12 months” [4].

The classification, severity, pathogenesis, and impact on daily life and health of disabled individuals are complex and plentiful, it would be impossible and illogical to resort to a uniform and simplified standard to measure their health, functioning, and life quality. The WHO supports that a complete and comprehensive assessment of all aspects of the definition would require a detailed clinical evaluation of the underlying medical cause(s) for the impairment; analysis of the expected duration of the impairment (prognosis); a comprehensive assessment of the work-related functional limitations attributable to the impairment, the individual’s remaining functional capacity; a detailed vocational analysis of the individual’s work history and acquired work skills, educational background, and age; and a thorough analysis of the individual’s current vocational prospects [8,9].

Clinicians use different assessment tools depending on the type and position of disability. In these tools, the patient-reported outcome measures (PROMs) are ideal methods to assess the specific functional abilities and life quality of the disabled. There are a lot of PROMs tools used in different kinds of diseases, impairments, disorders, and disabilities such as the Oswestry Disability Questionnaire, the Short Form 36 (SF-36), the Roland Morris Disability Questionnaire (RMDQ) [10,11,12,13,14,15,16,17,18,19,20,21], the Disability of the Shoulder, Arm, and Hand (DASH) Questionnaire, the SF-36 Health Survey Questionnaire, the Health Assessment Questionnaire [22,23,24,25], the American Academy of Orthopedic Surgeons lower limb questionnaire [26], the Lower-Limb Tasks Questionnaire [27], the Visual Analogue Scale (VAS), the Pain Diary [12,28,29,30,31,32], and the Functional Capacity Evaluation (FCE) [33]. All the reliability and responsiveness of these assessment tools had already been verified by previous studies which demonstrated that measurements obtained with these scales and questionnaires were reliable and had sufficient width scale to reliably detect improvement or worsening in most disabled [34,35,36,37,38,39,40].

Most disabilities are irreversible so their negative effects are not only detrimental for physical health but also for psychological health and social functioning. Therefore, treatment programs for disabled individuals should be aimed at more than improving their physical functions. Treatments programs for disabled individuals would be diverse, trying to improve their life quality from more perspectives, such as physical health, psychological health, and social functioning. For example, the psychological activity groups that used art activities were found to increase psychological well-being and satisfaction with life among the families of disabled children with various types of distress (physical, psychological, economic, and social), as well as reducing their parents’ perceived caregiving burden [41]. Progressive resistance training reduced self-reported difficulties in community-dwelling old persons with hip fractures sustained on average three years earlier, the effect could last for even several years after fracture [42]. A review by Singh in 2020 claimed that evidence supported the use of informal mindfulness practices for challenging behaviors of people with an intellectual and developmental disability (IDD) [43].

Since the variety of disabilities and the PROMs tools, the traditional pair-wise meta-analysis could not make a mixed comparison of different disabled-care programs from different perspectives. Moreover, there is no research pooled the effects of disabled-care programs systematically by using the results of the PROMs, the hardest part of which is to reclassify the interventions and the results of the PROMs into a class from a certain perspective or disciplinary. The bottleneck of this work is to reclassify the different scales and questionnaires of the PROMs according to their items and to normalize the results of these scales and questionnaires with different total scores through a reasonable mathematical method.

To solve this problem, this systematic review and network meta-analysis used the patient-reported outcome measures (PROMs) as the primary outcome measure, reclassifying different interventions into drug treatment, placebo treatment, usual care, active exercise, passive therapy, psychological education, psychosocial support programs, and multi-disciplinary programs, and reclassifying different scales and questionnaires of the PROMs into 4 different perspectives which were overall life quality, abilities of daily life activity, psychological health, and social functioning. At the same time, every score of the scale or questionnaire of the PROMs was transferred into the percentage of its total score so that all the outcomes could be uniformized into the same scale. The network meta-analysis, which is based upon the Bayes’ theorem, was used to make the mixed and indirect comparisons of the interventions, aiming to pool the effects of different disabled-care programs together. The objective of this review is to compare the effects of interventions from different perspectives and finally to make adjusted indirect and mixed comparisons of interventions for the patient-reported outcomes measures (PROMs) of disabled adults.

## 2. Methods

### 2.1. Protocol and Registration

This review was conducted according to the Preferred Reporting Items for Systematic Reviews and Meta-Analysis (PRISMA) guidelines. Literature eligibility and exclusion criteria and search strategy were proposed and agreed on by two authors (Y.X and X.L) and had been established a priori to minimize bias. The Registration Number of this review is CRD42021232058.

### 2.2. Eligibility Criteria (PICOS)

Since the theory-driven approach of the PROMs was made in 2004 [44] by using two sociological studies in 1995 and 1998 [45,46], and most scales of the PROMs were developed, revised, or widely used in the clinic after 1990, excluding studies before 1990 might reduce the publication bias of this systematic review. Therefore, studies published from 1990 to 2020 and randomized designed would be eligible for inclusion. The study must have been peer-reviewed and published in English. The PICOS of this review was as follows:

#### 2.2.1. Participants/Population

All studies whose participants were adults above 18 years old with disabilities would be included in this review. The definition of disability was following the description given by the SSA, which was that “the inability to engage in any substantial, gainful activity because of a medically determinable physical or mental impairment(s), which could be expected to result in death or which has lasted or can be expected to last for a continuous period of not less than 12 months” [4].

#### 2.2.2. Intervention(s)

As the principle of the re-classification of interventions, all the interventions in the included studies would be classified into eight classes: (1) Passive therapy (all kinds of physiotherapist-led interventions such as vibration therapy, massage, and passive stretching); (2) Active exercise (resistance training, exercise with or without supervision or assistance); (3) Usual care (treatment as usual, waiting list, and no-treatment); (4) Drug treatment; (5) Placebo; (6) Psychosocial support programs (service dogs, caregiver education programs, and group sessions); (7) Psychological education (cognitive behavior therapy); (8) Multi-disciplinary Programs (long-term group courses, multi-modal therapy, and psychological education combined with physical exercise).

#### 2.2.3. Comparator(s)/Control

Since the network meta-analysis is based upon the Bayes’ theorem, it is feasible to make the indirect comparisons of the interventions mentioned above. The comparator(s)/control criteria were the same as the intervention(s) criteria.

#### 2.2.4. Outcomes

The patient-reported outcome measures (PROMs) had been used as the primary outcome measure of this review. There are a lot of scales and questionnaires in PROMs. In this review, the self-reported scales and questionnaires had been re-classified into perspectives as follows: (1) Abilities of daily life activity; (2) Psychological health; (3) Social functioning; (4) Overall life quality. All the outcomes should be presented as scores, and the scales or questionnaire must have a total score. If not, the original scores could not be transferred into a uniform scale.

#### 2.2.5. Study Design(s)

To make sure that the evidence quality of this systematic review would be higher as possible, only studies of randomized controlled trials (RCTs), whether single-armed or multi-armed, would be included in this review.

### 2.3. Exclusion Criteria

Studies would be excluded if: (1) not all the participants in the study meet the SSA’s description of disability such as children, teenagers, and people with preclinical disabilities; (2) the study evaluated other treatments that could not be classified into one of the classes mentions in the PICOS, such as surgery and injections; (3) the study was a published abstract or lack of data; (4) the outcomes of the study could not be classified into one of the classes in the PICOS; (5) the scores of the outcome could be transferred into a percentage of a total score, for example, the outcome whose scores presented in exponential form would be excluded in this review; (6) the study was not about randomized controlled trials, such as cross-sectional studies, case reports, cohort studies and cross-over trials in a single group.

### 2.4. Search Strategy

A comprehensive, reproducible search strategy was performed on the following databases from 1 January 1990 to 31 December 2020: PubMed, MedLine, Ovid, and CINAHL. Reference lists of included studies were also searched. Grey literature was searched to identify potential studies. If data were insufficient, authors would be contacted and requested for missing data. The search terms used in each database was as follows: (1) in PubMed, the search term was “((people with disabilities) OR (disabled) OR (people with disability) OR (handicapped) OR (disable people) [Titile]) AND ((randomized) OR (randomised) [Title/Abstract])”; (2) in MedLine, CINAHL, and Ovid, the search term was “(TI people with disabilities OR disabled OR people with a disability OR handicapped OR disable people) AND ( AB randomized OR randomised) NOT (TI design or protocol or review)”.

The search term “disability*” was not used in the search strategy to reduce the heterogeneity within studies and increase the consistency of the mathematical model. The reason was that the results of the search term “disability*” would involve a lot of disorders, dysfunctions, and impairments caused by diseases that were completely curable or self-limited. These disabilities might not match the description given by the SSA, which was that “be expected to result in death or which has lasted or can be expected to last for a continuous period of not less than 12 months”.

Title, abstract, and full-text screening were made by two independent authors (Yining Xu and Xin Li). Any disagreement would be resolved by a third independent reviewer (Zhihong Sun).

### 2.5. Risk of Bias Assessment

The Cochrane Collaboration Risk of Bias Assessment Tool was used to evaluate the risk of bias. All the included studies were evaluated by two independent authors (Yining Xu and Xin Li). Any disagreement would be discussed and an independent arbitrator (Zhihong Sun) was invited when an agreement could not be met.

### 2.6. Data Extraction and Synthesis

All potential studies were imported into EndNote X9 (Thomson Reuters, Carlsbad, CA, USA) and duplicates would be removed. Data were extracted by two independent authors (Yining Xu and Xin Li). Any discrepancies would be solved by an independent arbitrator (Zhihong Sun).

Findings were summarized and population characteristics such as age, gender, type of disability, information of intervention protocols, and the reclassification of the intervention were collected and put into the extraction sheet of summary of included studies.

Details of the patient-reported outcome measures (PROMs), which included the full name, total score, length of follow-up, and reclassification of each scale, would be shown in another extraction sheet.

The original data of each study, which involved the sample size, the average scores within each group before and after the intervention, and the standard deviations of the scores, would be recorded in an independent extraction sheet for the data pre-processing. 

### 2.7. Data Pre-Processings

Data pre-processing and analysis were made by two independent investigators (Yining Xu and Xin Li). The Microsoft Office Excel (Version 16.0, Microsoft Corporation, Redmond, WA, USA) was used to pre-process the original data, transferring all the scores (mean ± SD) of different scales and questionnaires in the included studies into the form of a percentage of the total score (mean% ± SD%). The Aggregate Data Drug Information System (ADDIS V1.16.8 Produced by Drugis.org) was used to analyze all the processed data, calculated effect size, pool data into network meta-analysis, and output all the results and figures. The effect would be presented by the form of Mean differences (MD). The results of the network meta-analysis from each perspective would be presented in the following parts.

#### 2.7.1. Processing of a PROM with Subscales of Different Dimensions

If a PROM is a comprehensive measure of life quality with different subscales of various dimensions, each subscale would be considered separately and re-classified into a certain perspective. For example, the MOS 36-item short-form health survey (SF-36) would be divided into two components, a physical component score (SF-36 PCS) that belonged to a measure for Abilities of daily life activity, and a mental component score (SF-36 MCS) that belonged to a measure for psychological health.

#### 2.7.2. Processing of Different PROMs of the Same Dimension in a Study

If a study reported more than one PROM from the same perspective, the average score and its standard variation would be calculated. For example, the study by Szantan [47] reported scores of Activities of Daily Life Difficulty (ADL) and Instrumental Activities of Daily Life Difficulty, since both of two scales were PROMs for abilities of daily life activity, the average score of the two scales and its standard variation would be calculated. By this method, each study would have only one pair of data from each dimension before being pooled together into the network meta-analysis. Meanwhile, all the scores would be converted into positive (higher is better) by the method of converting the score of a negative scale (lower is better) into its opposite number.

### 2.8. Network Meta-Analysis

#### 2.8.1. Network Geometry

The network geometries displayed the overall number and type of treatments in comparison, informed indirectly by the Bayesian simulation modeling, and provided key information about the strength of evidence informing each direct link between two different treatments [48]. In each network geometry, every node represented one of the competing interventions, while the lines corresponded to the available direct comparisons between each pair of interventions, and the amount of available information could be presented by “weight” the edge using numbers of arms on them.

#### 2.8.2. Consistency and Inconsistency Analysis

If there are closed loops in the evidence structure, the inconsistency of the evidence should be assessed because in network meta-analysis the evidence structure is more complex. Inconsistency assessment could occur when a treatment C has a different effect when it is compared with A or B, for example, studies comparing A and C are systematically different from studies comparing B and C. Therefore, inconsistency may even occur with normal meta-analysis, but can only be detected using a network meta-analysis [49]. 

If there is no relevant inconsistency in the evidence, or there is no closed loop in the evidence structure, a consistency model could be used to conclude the relative effect of the included treatments. Network meta-analysis gives a consistent, integrated picture of the relative effects. However, given such a consistent set of relative effect estimates, it may still be difficult to conclude a potentially large set of treatments. Fortunately, the Bayesian approach makes it possible to process complex data, to estimate the probability that given by the priors and the data. The results would be shown in the rank probability plot. The sum of all rank probabilities is 1, both within a rank over treatments and within a treatment over ranks [49].

The valid results from network meta-analysis depended on the evidence network being internally consistent: direct and various sources of indirect evidence should be in agreement. Inconsistency referred to differences between direct and indirect effect estimates for the same comparison and significant inconsistency threatened the validity of the results of a network meta-analysis. Therefore, if presented, further exploration of inconsistency would be needed to identify possible sources of disagreement [50]. The random-effects standard deviations would be calculated under both consistency and inconsistency models and compared with each other to identify if there was inconsistency within interventions. If random effects standard deviations calculated under both consistency and inconsistency models were fully identical, it meant that there was a good consistency with the interventions. If not, the *p*-values from the analysis of the node splitting would be checked to determine which modal would be used [51].

#### 2.8.3. Network Meta-Analysis

A league table would be after the model of data analysis had been determined, reporting results that represented the mean difference with 95% confidence intervals in the column-defining treatment compared with the row-defining treatment [52]. 

If the included studies had a good consistency, the ranking of measures and probability would be made to facilitate simultaneous inference regarding all treatments. A table showing the ranking of treatments would be made, based on the probability of each treatment being the most effective or the least effective. The overall sum of the percentage in each row or column should be 1.00 (100%) [53]. Probabilities are estimated for a treatment to be ranked at a specific place (first, second, and so on) according to each outcome. However, a ranking of treatments based solely on the probability for each treatment of being the best should be avoided. This is because the probability of being the best does not account for the uncertainty in the relative treatment effects and can spuriously give higher ranks to treatments for which little evidence is available. The probability of being the best has the disadvantage that it does not reflect the spread of rankings for the treatments and to consider just the crude figures may be misleading [54,55].

### 2.9. Additional Analysis

#### 2.9.1. Pair-Wised Meta-Analysis

If two interventions were appearing separately, an additional pair-wise meta-analysis should be made. The result would be shown in forest-plot and the heterogeneity within studies would be assessed by the statistic I^2^ [56].

#### 2.9.2. The Split Note Calculation

While the results are easier to interpret, it requires a separate model to be run for each node to be split [49]. The node-splitting analysis is an alternative method to assess inconsistency in network meta-analysis. It assesses whether direct and indirect evidence on a specific node (the split node) is in agreement.

Node splitting has been proposed by Dias et al. [49] and essentially involves distinguishing between the direct and indirect evidence. It aims to identify consistency discrepancies associated with specific nodes. It is performed within a Bayesian framework and is computationally more intensive than other approaches. Whether the identified discrepancy is statistically significant could be determined by examining the calculating a respective Bayesian *p*-value [49].

## 3. Results

### 3.1. Search Strategy and Information Extraction

**Figure 1:**
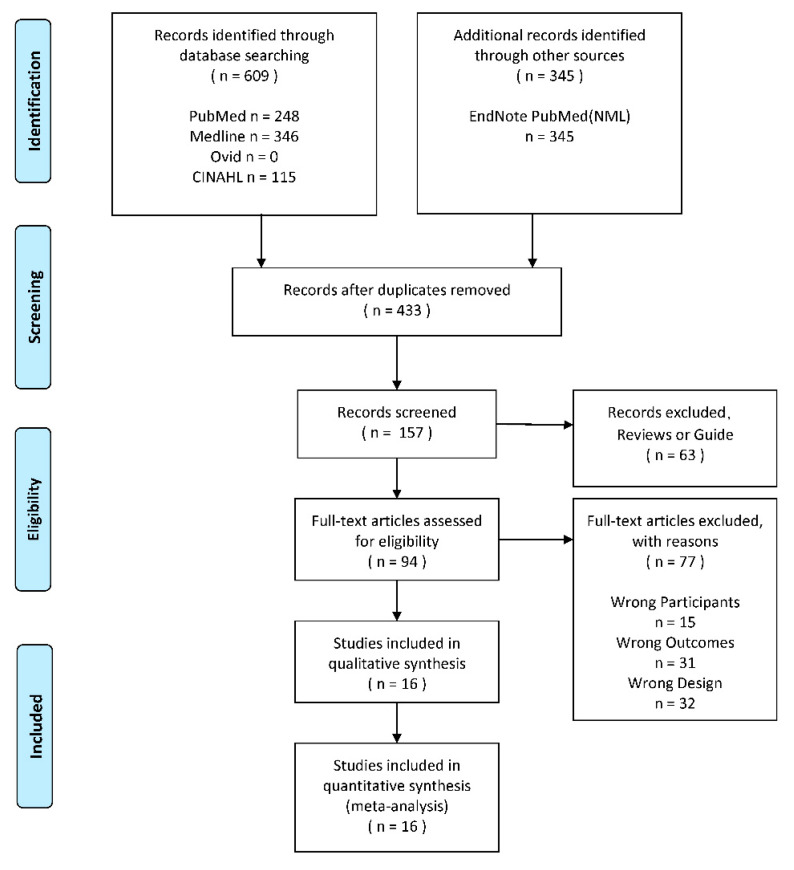
The PRISMA 2009 flow diagram of search and study selection.

The search yielded 954 titles and abstracts for screening. 94 full texts were screened and 14 were excluded. Sixteen studies were included in the final analysis [47,57,58,59,60,61,62,63,64,65,66,67,68,69,70,71]. The identification process is shown by a flow diagram [72] (Figure 1). The information of all included studies is shown in Table 1, and all the information about the PROMs was provided in Table 2. All the original data is shown in the Supplementary file.

### 3.2. Risk of Bias

**Figure 2:**
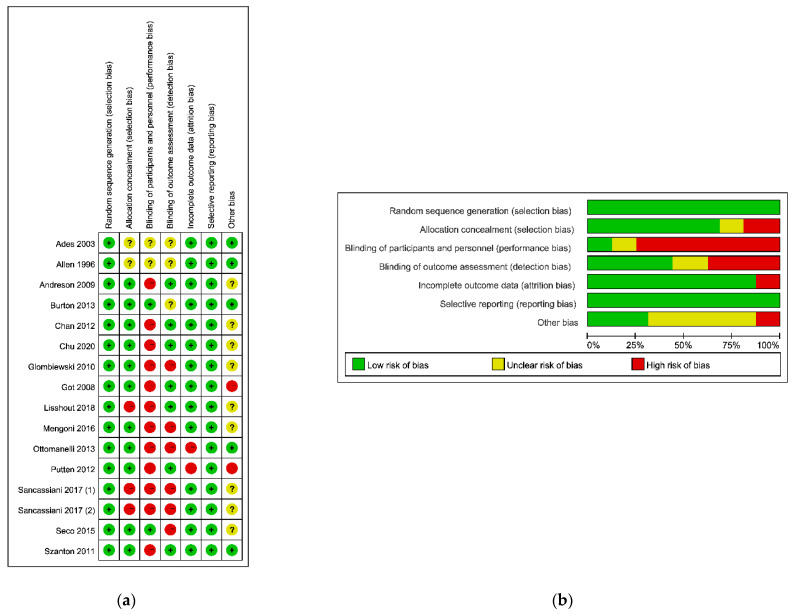
The result of the risk of bias assessment. (**a**) Risk of bis summary; (**b**) Risk of bias graph.

The result of the risk of bias assessment is shown in Figure 2. After discussion, a consensus was obtained for all items. Overall results were shown in Figure 2a. It could be seen that four studies had a high risk of bias, four studies had a moderate risk of bias, and eight studies had a low risk of bias. The overall bias was presented in Figure 2b: (1) the risk of performance bias (blinding of participants and personnel) was high (high in 12 studies ); (2) the risk of detection bias (blinding of outcome assessors) was low (high in six studies); (3) the risk of attrition bias (incomplete outcome data) was low (high in two studies); (4) the risk of selection bias (random sequence generation and allocation concealment) was low (high in three studies); (5) the risk of reporting bias (selective reporting of outcomes) was low (low in all studies).

### 3.3. Network Meta-Analysis

#### 3.3.1. Overall Life Quality

**Figure 3:**
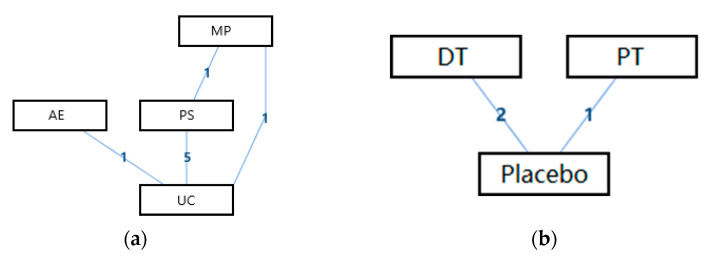
The network geometry of the interventions for the overall life quality of the disabled. (**a**) The mixed treatments comparison of AE, MP, PS, and UC; (**b**) The adjusted indirect comparison of DT, PT, and Placebo (AE: Active Exercise; MP: Multi-disciplinary Program; PS: Psychosocial Support Program; UC: Usual Care; DT: Drug Treatment; PT: Passive Therapies).

The network geometry of the interventions for the overall life quality of the disabled was presented in Figure 3. From this perspective, there was a mixed interventions comparison of AE, MP, PS, and UC (Figure 3a), and there was an adjusted indirect comparison of interventions of DT, PT, and Placebo (Figure 3b). Since there was a closed loop in the evidence structure, the inconsistency of the evidence should be assessed. 

In the mixed treatments comparison of AE, MP, PS, and UC, the random effects standard deviations of the consistency modal and its 95% confidence intervals were 0.02 (0.00, 0.07), the random effects standard deviations of the inconsistency modal and its 95% confidence intervals were 0.03 (0.00, 0.07), and the inconsistency standard deviation of the inconsistency modal and its 95% confidence intervals were 0.04 (0.00, 0.07). Moreover, the inconsistency factors with the 95% confidence intervals in the cycle of MP, PS, and UC were −0.00 (−0.10, 0.08). The mean value of the inconsistency factors was closed to 0. Therefore, there might be consistency discrepancies associated with specific nodes, and that it was necessary to make a node splitting analysis.

In the adjusted indirect comparison of DT, PT, and Placebo, the random effects standard deviations of the consistency modal and its 95% confidence intervals were 0.55 (0.24, 0.80), the random effects standard deviations of the inconsistency modal and its 95% confidence intervals were 0.55 (0.24, 0.80), and the inconsistency standard deviation of the inconsistency modal and its 95% confidence intervals were also 0.42 (0.02, 0.79). Since the random effects standard deviations of the consistency modal and the inconsistency modal were almost the same. It means that the analysis under consistency modal had a good validity.

Table 3 shows the league tables of the network geometries in Figure 3a,b. Bold characters indicate that the data was statistically significant (0 was not included in the 95% confidence intervals).

**Figure 4:**
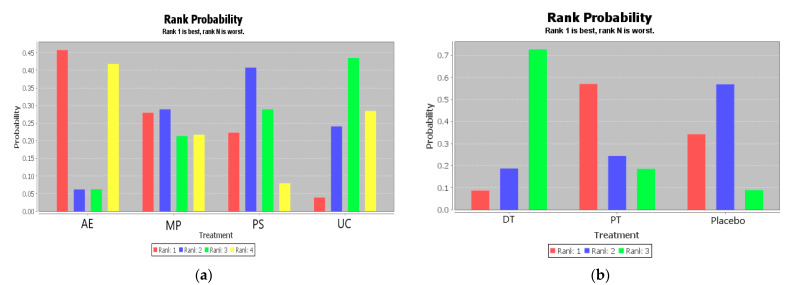
The ranking of measures and probabilities of the interventions for the overall life quality of the disabled. (**a**) adjusted indirect comparison of AE, MP, PS, and UC; (**b**) adjusted indirect comparison of DT, PT, and Placebo (AE: Active Exercise; MP: Multi-disciplinary Program; PS: Psychosocial Support Program; UC: Usual Care; DT: Drug Treatment; PT: Passive Therapies).

The ranking of measures and probabilities is provided in Table 4 and shown as a bar graph (Figure 4). What should be paid attention to was the fact that since the smaller hallux valgus angle indicated a better condition, in the figure of rank probability, Rank 1 was the best one, and Rank N was the worst one. According to the results, Active Exercise and Passive Therapy might have the highest probability of being the best intervention for the overall life quality of the disabled. 

Since there was no pair of two interventions appearing separately, it was unnecessary to perform a pair-wise meta-analysis. The results of the node splitting analysis would be provided in Table 5, which showed the estimated quantiles for the direct evidence, the indirect evidence, the combined evidence, as well as the *p*-value. A large *p*-value indicates no significant inconsistency was found. According to Table 5, all the *p*-values were greater than 0.05, meaning that the consistency model should be used. 

#### 3.3.2. Abilities of Daily Life Activity

**Figure 5:**
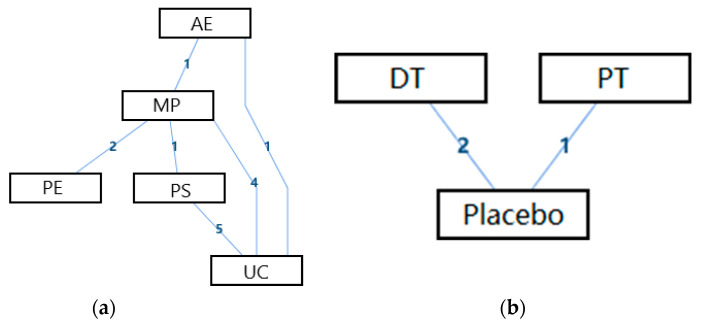
The network geometry of the interventions for abilities of daily life activity of the disabled. (**a**) The mixed treatments comparison of AE, MP, PE, PS, and UC; (**b**) The adjusted indirect comparison of DT, PT, and Placebo (AE: Active Exercise; MP: Multi-disciplinary Program; PE: Psychological Education; PS: Psychosocial Support Program; UC: Usual Care; DT: Drug Treatment; PT: Passive Therapies).

The network geometry of the interventions for the abilities of daily life activity of the disabled is presented in Figure 5. From this perspective, there was a mixed interventions comparison of AE, MP, PE, PS, and UC (Figure 5a), and there was an adjusted indirect comparison of interventions of DT, PT, and Placebo (Figure 5b). Since there was a closed loop in the evidence structure, the inconsistency of the evidence should be assessed. 

In the mixed treatments comparison of AE, MP, PE, PS, and UC, the random effects standard deviations of the consistency modal and its 95% confidence intervals were 0.01 (0.00, 0.03), the random effects standard deviations of the inconsistency modal and its 95% confidence intervals were 0.01 (0.00, 0.03), and the inconsistency standard deviation of the inconsistency modal and its 95% confidence intervals were 0.07 (0.00, 0.18). Moreover, the inconsistency factors with the 95% confidence intervals in the cycle of MP, PS, and UC were −0.01 (−0.20, 0.10), and the inconsistency factors with the 95% confidence intervals in the cycle of AE, MP, PS, and UC were −0.01 (−0.16, 0.10). The mean value of the inconsistency factors of the two cycles were both closed to 0. Therefore, there might be consistency discrepancies associated with specific nodes, and that it was necessary to make a node splitting analysis. 

In the adjusted indirect comparison of DT, PT, and Placebo, the random effects standard deviations of the consistency modal and its 95% confidence intervals were 0.01 (0.00, 0.03), the random effects standard deviations of the inconsistency modal and its 95% confidence intervals were 0.02 (0.00, 0.03), and the inconsistency standard deviation of the inconsistency modal and its 95% confidence intervals were also 0.01 (0.00, 0.03). Since the random effects standard deviations of the consistency modal and the inconsistency modal were almost the same. It means that the analysis under consistency modal had a good validity.

Table 6 shows the league tables of the network geometries (Figure 5a,b). Bold characters indicated that the data was statistically significant (0 was not included in the 95% confidence intervals).

**Figure 6:**
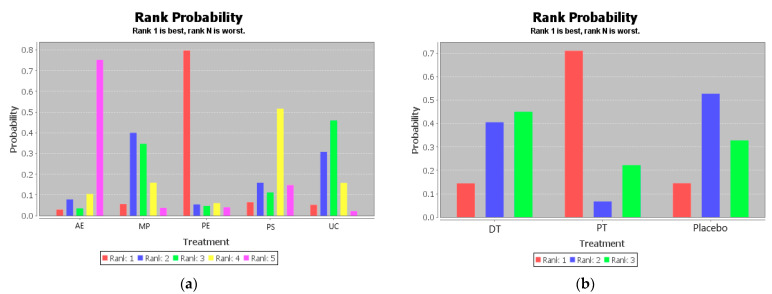
The ranking of measures and probabilities of the interventions for abilities of daily life activity of the disabled. (**a**) Adjusted indirect comparison of AE, MP, PE, PS, and UC; (**b**) adjusted indirect comparison of DT, PT, and Placebo. (AE: Active Exercise; MP: Multi-disciplinary Program; PE: Psychological Education; PS: Psychosocial Support Program; UC: Usual Care; DT: Drug Treatment; PT: Passive Therapies).

The ranking of measures and probabilities would be provided in Table 7 and shown in the bar graph (Figure 6). What should be paid attention to was that since the smaller hallux valgus angle indicated a better condition, in the figure of rank probability, Rank 1 was the best one, and Rank N was the worst one. According to the results, Psychological Education and Passive Therapy might have the highest probability of being the best intervention for the abilities of daily life activity of the disabled. 

Since there was no pair of two interventions appearing separately, it was unnecessary to make a pair-wise meta-analysis. The results of the node splitting analysis would be provided in Table 8, which showed the estimated quantiles for the direct evidence, the indirect evidence, the combined evidence, as well as the *p*-value. A large *p*-value indicates no significant inconsistency was found. According to Table 8, all the *p*-values were greater than 0.05, meaning that the consistency model should be used. 

#### 3.3.3. Psychological Health

**Figure 7:**
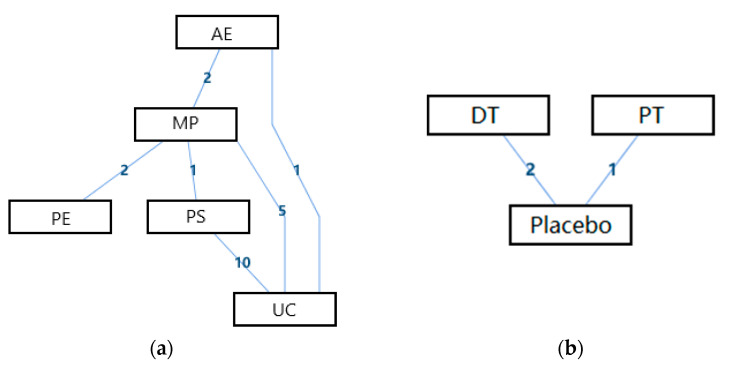
The network geometry of the interventions for the psychological health of the disabled. (**a**) The mixed treatments comparison of AE, MP, PE, PS, and UC; (**b**) The adjusted indirect comparison of DT, PT, and Placebo (AE: Active Exercise; MP: Multi-disciplinary Program; PE: Psychological Education; PS: Psychosocial Support Program; UC: Usual Care; DT: Drug Treatment; PT: Passive Therapies).

The network geometry of the interventions for the psychological health of the disabled was presented in Figure 7. From this perspective, there was a mixed interventions comparison of AE, MP, PE, PS, and UC (Figure 7a), and there was an adjusted indirect comparison of interventions of DT, PT, and Placebo (Figure 7b). Since there was a closed loop in the evidence structure, the inconsistency of the evidence should be assessed.

In the mixed treatments comparison of AE, MP, PE, PS, and UC, the random effects standard deviations of the consistency modal and its 95% confidence intervals were 0.31 (0.23, 0.47), the random effects standard deviations of the inconsistency modal and its 95% confidence intervals were 0.32 (0.23, 0.48), and the inconsistency standard deviation of the inconsistency modal and its 95% confidence intervals were 0.24 (0.01, 0.72). Moreover, the inconsistency factors with the 95% confidence intervals in the cycle of MP, PS, and UC were 0.00 (−0.45, 0.47), and the inconsistency factors with the 95% confidence intervals in the cycle of AE, MP, PS, and UC were −0.01 (−0.58, 0.53). The mean value of the inconsistency factors of the two cycles were both closed to 0. Therefore, there might be consistency discrepancies associated with specific nodes, and that it was necessary to make a node splitting analysis.

In the adjusted indirect comparison of DT, PT, and Placebo, the random effects standard deviations of the consistency modal and its 95% confidence intervals were 0.01 (0.00, 0.03), the random effects standard deviations of the inconsistency modal and its 95% confidence intervals were 0.01 (0.00, 0.03), and the inconsistency standard deviation of the inconsistency modal and its 95% confidence intervals were also 0.02 (0.00, 0.03). Since the random effects standard deviations of the consistency modal and the inconsistency modal were almost the same. It means that the analysis under consistency modal had a good validity.

Table 9 shows the league tables of the network geometries (Figure 7a,b). Bold characters indicated that the data was statistically significant (0 was not included in the 95% confidence intervals).

**Figure 8:**
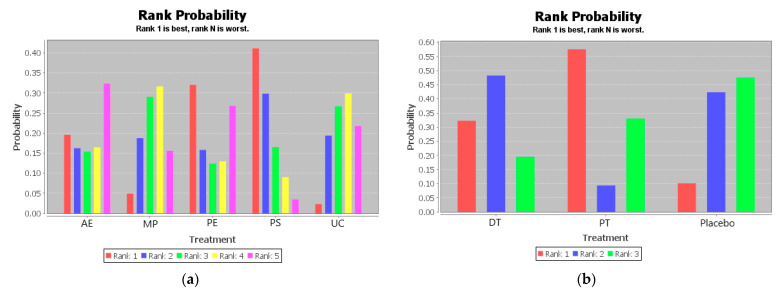
The ranking of measures and probabilities of the interventions for the psychological health of the disabled. (**a**) Adjusted indirect comparison of AE, MP, PE, PS, and UC; (**b**) adjusted indirect comparison of DT, PT, and Placebo (AE: Active Exercise; MP: Multi-disciplinary Program; PE: Psychological Education; PS: Psychosocial Support Program; UC: Usual Care; DT: Drug Treatment; PT: Passive Therapies).

The ranking of measures and probabilities is provided in Table 10 and shown as a bar graph (Figure 8). What should be paid attention to was that, since the smaller hallux valgus angle indicated a better condition, in the figure of rank probability, Rank 1 was the best one, and Rank N was the worst one. According to the results, the Psychosocial Support Program and Passive Therapy might have the highest probability of being the best intervention for the psychological health of the disabled. 

Since there was no pair of two interventions appearing separately, it was unnecessary to make a pair-wise meta-analysis. The results of the node splitting analysis would be provided in Table 11, which showed the estimated quantiles for the direct evidence, the indirect evidence, the combined evidence, as well as the *p*-value. A large *p*-value indicates no significant inconsistency was found. According to Table 11, all the *p*-values were greater than 0.05, meaning that the consistency model should be used.

#### 3.3.4. Social Functioning

**Figure 9:**
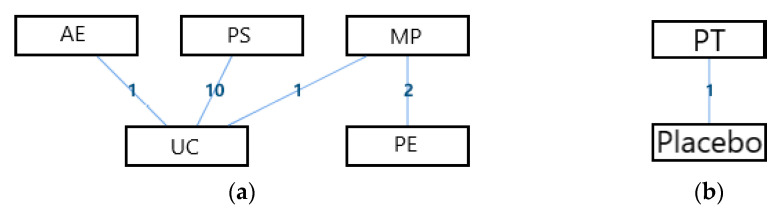
The network geometry of the interventions for the social functioning of the disabled. (**a**) The adjusted indirect comparison of AE, PS, MP, PE, and UC; (**b**) The direct comparisons of PT and Placebo (AE: Active Exercise; MP: Multi-disciplinary Program; PE: Psychological Education; PS: Psychosocial Support Program; UC: Usual Care; DT: Drug Treatment; PT: Passive Therapies).

The network geometry of the interventions for the social functioning of the disabled was presented in Figure 9. There was an adjusted indirect comparison of interventions (Figure 9a) and a directed comparison of interventions (Figure 9b) from this perspective. There was no closed loop in the evidence structure, so a consistency model would be used to conclude the relative effect of the included treatments. 

Before using the consistency modal, the vilification of the modal would be done. In the adjusted indirect comparison of AE, PS, MP, PE, and UC, the random effects standard deviations of the consistency modal and its 95% confidence intervals were 0.36 (0.23, 0.64), the random effects standard deviations of the inconsistency modal and its 95% confidence intervals were 0.36 (0.24, 0.63), and the inconsistency standard deviation of the inconsistency modal and its 95% confidence intervals were also 0.41 (0.02, 0.80). Since the random effects standard deviations of the consistency modal and the inconsistency modal were almost the same. It means that the analysis under consistency modal had a good validity. 

Table 12 shows the league tables of the network geometries (Figure 9a). Bold characters indicated that the data was statistically significant (0 was not included in the 95% confidence intervals).

**Figure 10:**
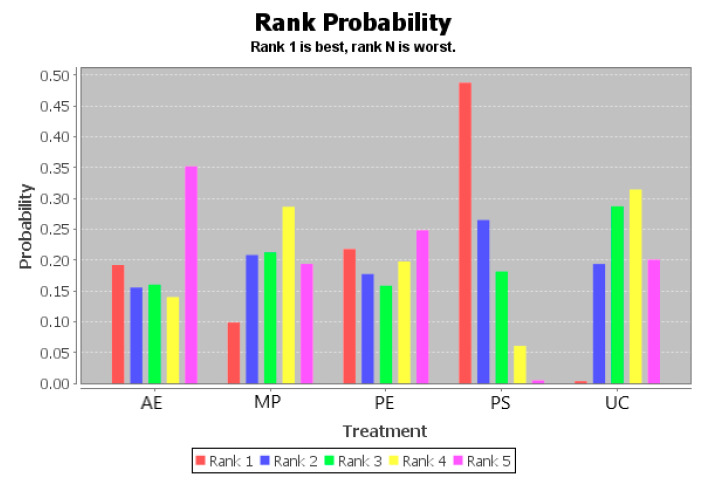
The ranking of measures and probabilities of the interventions for the social functioning of the disabled (AE: Active Exercise; MP: Multi-disciplinary Program; PE: Psychological Education; PS: Psychosocial Support Program; UC: Usual Care).

The ranking of measures and probabilities is provided in Table 13 and shown as a bar graph (Figure 10). What should be paid attention to was that, since the smaller hallux valgus angle indicated a better condition, in the figure of rank probability, Rank 1 was the best one, and Rank N was the worst one. According to the results, the Psychosocial Support Program might have the highest probability of being the best intervention for the social functioning of the disabled. 

**Figure 11:**
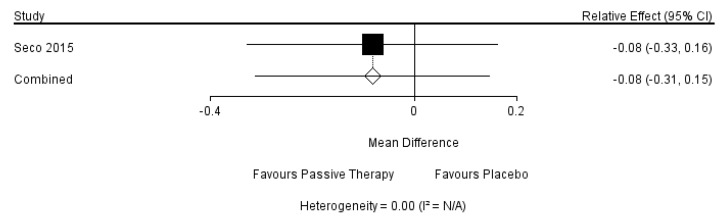
The forest plots of the direct comparisons of interventions.

The results of the direct comparison of interventions would be provided by forest plots as shown in Figure 11. Moreover, since the results could not be interpreted, it wasn’t required a separate model to be run for each node to be split.

## 4. Discussion

PROMs have an extremely relevant role in practice. Managing to implement a systematic collection of PROMs would be one of the hardest challenges at a system level. The collection of PROMs may become part of clinicians’ daily practice and may lead to a change in the relationship and communication between clinicians and their patients. By this say, clinicians could accept to have their job reviewed and not be afraid to be evaluated by their patients [73]. Introducing a successful systematic collection of PROMs would be beneficial for the performance of clinicians, improve the patients’ satisfaction, and provide more valuable information for the development of disabled-care programs. Further research should be helpful for the managers of the medical system and the social security to formulate an official guide for collecting PROMs of the disabled systematically.

The results show that, to overall life quality, active exercise and passive therapy might have the most potential to becoming the best choice of intervention., Disability would cause inability to engage in any substantial, gainful activity and then decrease the daily life activities of the disabled because of a medically determinable physical or mental impairment. With the decrease of daily activity, the self-efficacy and activity willingness of the disabled also decrease gradually [74]. Therefore, it would be most important for the disabled to preserve their remaining physical functions as much as possible so that they could keep their physical activities as more as possible. However, disabilities might increase the risk of injury or re-injury when people with disabilities doing active exercises [75]. The fear of injury and re-injury would make disabled people suffering from psychological setbacks and fear-avoidance beliefs during training. It indicated that future research needs to explore the best active exercise or passive therapy scheme for the disabled.

As to the abilities of daily life activity, there are no statistically significant differences in all the head-to-head comparisons. However, the strengths of the network meta-analysis, which based on the Bayesian method, lies in that the significance of difference and the *p*-value would no longer be the main factor affecting the conclusion, and the intervention that is most likely to be the best choice could be selected by probability judgment. According to the result presented in the table of the ranking of measures and probabilities, psychological education and passive therapy are the most potential interventions. The difference between the effects of psychological education and usual care is almost statistically significant. The reason might be that the outcomes in this review are Patient-reported scales or questionnaires which might show some kind of subjectivity, and the psychological education could increase the self-efficacy and self-esteem of people with disabilities [76,77,78] so that the patients might report positively. In a conclusion, it is undeniable that psychological interventions could successfully increase the subjective feelings of the disabled and make them more active in daily life and healthier [79].

When considering psychological health, the most potential intervention is a psychosocial support program, whose effect is statistically significant when compared with that of usual care. The result indicated that, since all the disabled are part of the society and improving the overall life quality of people with disabilities is essential to complete their socialization, psychosocial factors should be taken into serious consideration when designing a care program for the disabled and all sectors of the society should be involved [80,81,82,83]. Moreover, when designing a disabled-care program, the focus should not only be on the subjective psychological health of the disabled but also their psychosocial health. Different severity of disabilities might represent different residual body function, people with different disabilities would have different abilities of daily life activity. However, it doesn’t mean that people with poorer physical abilities necessarily have poorer psychosocial health. For example, completely paralyzed patients might have the same psychological health as patients with mild disabilities. In further researches, the correlation of psychological well-being, the willingness of activities, and abilities of daily life activity should be studied [84,85].

When it comes to social functioning, a psychosocial support program, whose effect is significantly different from that of usual care, is the most potential intervention. Seco’s team concluded that the effect of passive therapy was better than placebo [57]. However, there was no trial compared the effects of psychosocial support program and passive therapy directly, meaning that the result that the multi-disciplinary program which included psychosocial support is less potential than psychosocial support program in this network meta-analysis is possible because of the lack of data. Besides, the cost-efficiency of disabled-care programs is always the focus of social concern [86,87,88]. However, few studies are comparing the comprehensive cost-efficiency of disabled-care programs. Future studies should focus on the optimal application of resources, especially in multi-disciplinary programs.

The strengths of this systematic review are that, first of all, different outcomes of PROMs and interventions were reclassified so that, as showed in the results, the heterogeneity within studies is reduced, the consistency of the calculation model is increased, and the inconsistency indicators of each model are very low. Secondly, the scores of scales and questionnaires with different total scores are normalized into the same scale, making it feasible to pool the original data together and compare the results. Finally, the use of network meta-analysis realizes to make adjusted indirect and mixed comparisons of different types of interventions.

The main limitation of this systematic review is that all the disabilities caused by fully curable or self-limited diseases were excluded in this review. However, some diseases, both acute and chronic, could cause irreversible disabilities that meet the criteria of the SSA. Meanwhile, not every potential study reported the detail of the participants’ disabilities, making it infeasible to judge every disability in each potential study in the library with the criteria of the SSA. This limitation also illustrates the necessity to introduce a successful systematic collection of PROMs.

## 5. Conclusions

This systematic review reclassified the interventions for the disabled into active exercise, passive therapy, psychological education, psychosocial support program, multi-disciplinary program, and usual care, using the method of network meta-analysis to compare the effects of these interventions from the perspective of abilities of daily life activity, psychological health, social functioning, and overall life quality. Consistency modal was used in the network meta-analysis and had been verified a good consistency. In conclusion, active exercise and passive therapy are most likely to be the best choices for overall life quality, psychological education and passive therapy are most likely to be the best choices for abilities of daily life activity, and psychosocial support programs are most likely to be the best choice for psychological health and social functioning. The results remind us that the disabled are also an important part of society, intervention programs for the disabled should not only focus on their physical health but also their psychological health and socialization.

## Figures and Tables

**Table 1:** Information about the included studies.

Study	Participants			Intervention					
	Disability	Mean Age	Gender (Female/All)	Operate Group			Control Group		
				Intervention	Protocol	Classification	Intervention	Protocol	Classification
Ades 2003 [71]	Disabled older female cardiac patients	72.3	33/33	Resistance training	19 participants, 3 weekly, 6 months	Active exercise	Cardiac rehabilitation facility, a program of stretching, calisthenics, deep-breathing progressive-relaxation exercises, and light yoga	14 participants, 3 weekly, 6 months	Multi-disciplinary program
Andreson 2009 [69]	Physically disabled older people	84.0	35/50	Nursing home settings program	27 participants, 12 weeks	Multi-disciplinary program	Usual care and treatment	20 participants, 12 weeks	Usual care
Button 2013 [68]	Older People with a self-reported physical disability	74.6	55/120	Spironolactone	25 mg/day, 24 weeks	Drug treatment	Placebo	25mg/day, 24 weeks	Placebo
Chan 2012 [67]	People with poststroke disability	69.1	2/14	Yoga and exercise program	1 weekly group yoga, 4 weekly home exercise, 6 weeks	Multi-disciplinary program	Exercise	Self-reported home practice	Active exercise
Chu 2020 [66]	Disabled stroke patients	64.5	37/61	Caregiver education program	Rehabilitation training, self-care, and toileting delivered, post-discharge telephone calls	Psychosocial support program	Usual care	Usual care	Usual care
Glomebiewski 2020 [65]	Severely disabled, chronic back pain patients	48.8	77/116	Cognitive-behavioral therapy including biofeedback tools	An 18 sessions program including EMG feedback, 8 months	Multi-disciplinary program	Cognitive-behavioral therapy, 8 months	An 18 sessions program	Psychological education
Sancassiani 2017(1) [60]	People with severe psychosocial disabilities	37.2	11/51	Sailing course plus drugs treatments	A 3 months-lasting sailing course plus drug treatments as usual.	Multi-disciplinary program	Rehabilitation treatment as usual	Rehabilitation treatment as usual	Usual care
Seco 2015 [59]	Severely disabled patients	45.5	7/20	Vibration therapy	2 weekly, 8 weeks	Passive therapy	Placebo vibration therapy	2 weekly, 8 weeks	Placebo
Putten 2012 [58]	People with profound intellectual and multiple disabilities	32.1	Not mentioned	Power-assisted exercise	3 weekly, 20 weeks	Active exercise	Therapy as usual	Therapy as usual	Usual care
Ottomanelli 2013 [62]	Veterans with spinal cord injury	49.2	0/157	Support employment program	Employment support in 48 weeks	Psychosocial support program	Treatment as usual	Treatment as usual	Usual care
Lieshout 2018 [57]	Independently living older people with disability	74.0	155/281	A proactive multicomponent intervention program	Spry-program, 23 weeks	Multi-disciplinary program	Usual care	Usual care	Usual care
Allen 1996 [70]	People with severe ambulatory disabilities	Not mentioned	50/100	Service dogs	24 months staying with a service dog	Psychosocial support program	Waiting list	Waiting list	Usual care
Got 2008 [64]	People with a developmental disability	27.0	16/38	Art facilitation	12 group art-making sessions in 12 weeks	Psychosocial support program	No-treatment	No-treatment	Usual care
Mengoni 2016 [63]	People with epilepsy and learning disabilities	41.7	23/40	Wordless intervention	Using a picture booklet with a trained researcher and a caregiver present at least twice more over 20 weeks.	Psychosocial support program	Blank	Blank	Usual care
Szanton 2011 [47]	Disabled old people	78.2	38/40	Bio-behavior-environmental intervention	The community aging in place, advancing better living for elders, 10 in-home sessions, 24 weeks	Multi-disciplinary program	Attention and education control	Attention and education control	Psychosocial support program
Sancassiani 2017(2) [61]	People with severe psychosocial disabilities	37.2	11/51	Sailing course plus drugs treatments	A 3 months-lasting sailing course plus drug treatments as usual.	Multi-disciplinary program	Rehabilitation treatment as usual	Rehabilitation treatment as usual	Usual care

**Table 2:** Information about the PROMs extracted from the included studies.

Study	Patient-Reported Outcome Measures (PROM)			
	Scale	Total Score	Follow up (Weeks)	Classification
Ades 2003 [71]	Continous-Scale Physical Performance Test (CS-PFP)	100	0/24	Abilities of daily life activity
	MOS SF-36 Physical Functioning (SF 36-PF)	100		Abilities of daily life activity
Andreson 2009 [69]	The Measure of Actualization of Potential Test (MAP)	100	0/12/24	Abilities of daily life activity
Burton 2013 [68]	EuroQoL-Visual Analogue Scale (EQ-VAS)	100	0/10/20	Abilities of daily life activity
	EuroQoL- 5 Dimensions (EQ-5D)	1.59		Overall life quality
	The Function Limitation Profile (FLP)	117		Overall life quality
	Hospital Anxiety and Depression Scale-Depression (HADS-D)	21		Psychological Health
	Hospital Anxiety and Depression Scale-Anxiety (HADS-A)	21		Psychological Health
Chan 2012 [67]	The Geriatric Depression Scale-Short Form (GDS-15)	15	0/6	Psychological Health
	State-Trait Anxiety Inventory- State (STAT-S/Y1)	80		Psychological Health
	State-Trait Anxiety Inventory- Trait (STAT-T/Y2)	80		Psychological Health
Chu 2020 [66]	Caregiver Burden Inventory (CBI)	96	0/32	Social Functioning
	EuroQoL- 5 Dimensions (EQ-5D)	1.59		Overall life quality
	Barthel Index (BI)	100		Social Functioning
Glombiewski 2010 [65]	Pain Intensity Questionnaire (PIQ)	10	0/32/56	Abilities of daily life activity
	Pain Diary	10		Abilities of daily life activity
	Pain Disability Index (PDI)	7		Abilities of daily life activity
	Health-related Life Satisfaction Scale	35		Psychological Health
	Beck Depression Inventory (BDI)	63		Psychological Health
	Coping Strategies Scale (CSS)	112		Social Functioning
Sancassian 2017 (1) [60]	Clinical Global Impression-Severity Scale (CGI-S)	7	0/12	Abilities of daily life activity
	Biological Rhythms Interview of Assessment in Neuropsychiatry (BRAIN)	84		Overall life quality
	Global Assessment of Functioning (GAF)	100		Abilities of daily life activity
	The health of the Nation Outcome Scale - Total	48		Overall life quality
	The health of the Nation Outcome Scale—Behavioral	12		Social Functioning
	The health of the Nation Outcome Scale—Cognitive and physical impairment	8		Overall life quality
	The health of the Nation Outcome Scale—Psychopathological symptoms	12		Psychological Health
	The health of the Nation Outcome Scale—Social	16		Social Functioning
Sancassian 2017 (2) [61]	Sense of Coherence (SOC-13)	91	0/12	Psychological Health
	The short-form health survey—Physical component score (SF-12 PCS)	47		Abilities of daily life activity
	The short-form health survey—Mental component score (SF-12 MCS)	47		Psychological Health
	General self-efficacy scale (GSES)	40		Psychological HealthGot
Seco 2015 [59]	WHO Quality of Life -Physical Health	35	0/8	Abilities of daily life activity
	WHO Quality of Life -Psychological Health	30		Psychological Health
	WHO Quality of Life -Social Relationship	20		Social Functioning
	WHO Quality of Life -Environment	40		Social Functioning
	WHO Quality of Life -General Health	10		Overall life quality
	State-Trait Anxiety Inventory- State (STAT-S/Y1)	80		Psychological Health
Lieshout 2018 [57]	Groningen Frailty Indicator (GFI)	15	0/75	Abilities of daily life activity
	Quality of Life-Physical Composite Scale (SF 12-PCS)	50		Abilities of daily life activity
	Quality of Life-Mental Composite Scale (SF 12-MCS)	50		Psychological Health
Allen 1996 [70]	The Spheres of Control Scale (SCS)	180	0/24/48	Psychological Health
	Rosenberg Self-esteem Scale (RSS)	50		Psychological Health
	Affect Balance Scale (ABS)	9		Psychological Health
	Community Integrated Questionnaire	28		Social Functioning
Putton 2012 [58]	Behavioral Appraisal Scale (BAS)	100	0/20	Overall life quality
	Alertness Observation List	100		Psychological Health
	Quality of Life-PMD Physical Wellbeing	100		Abilities of daily life activity
	Quality of Life-PMD Material Wellbeing	100		Psychological Health
	Quality of Life-PMD Communication Wellbeing	100		Social Functioning
	Quality of Life-PMD Social Wellbeing	100		Social Functioning
	Quality of Life-PMD Development	100		Social Functioning
	Quality of Life-PMD Activities	100		Psychological Health
Got 2008 [64]	The scale of independent behavior—revised (SIB-R) Social interaction	5	0/12	Social Functioning
	The scale of independent behavior—revised (SIB-R) Language comprehension	5		Social Functioning
	The scale of independent behavior—revised (SIB-R) Language expression	5		Social Functioning
	Quality of life enjoyment and satisfaction questionnaire (Q-LES-Q) Subjective feelings	3		Psychological Health
	Quality of life enjoyment and satisfaction questionnaire (Q-LES-Q) Leisure time activities	3		Social Functioning
	Quality of life enjoyment and satisfaction questionnaire (Q-LES-Q) Social relationship	3		Social Functioning
	Quality of life enjoyment and satisfaction questionnaire (Q-LES-Q) General activities	3		Abilities of daily life activity
engoni 2016 [63]	Epilepsy and learning disabilities quality of life scale (ELDQOL)—Seizure severity	70	0/4/12/20	Abilities of daily life activity
	Epilepsy and learning disabilities quality of life scale (ELDQOL)—The side effect	95		Abilities of daily life activity
	Epilepsy and learning disabilities quality of life scale (ELDQOL)—Behavior	45		Social Functioning
	Epilepsy and learning disabilities quality of life scale (ELDQOL)—Mood	80		Psychological Health
	EuroQoL-Visual Analogue Scale (EQ-VAS)	100		Abilities of daily life activity
	EuroQoL- 5 Dimensions (EQ-5D)	1.59		Overall life quality
Ottomanelli 2013 [62]	Veterans RAND 36-item health survey (VR-36)—Physical component score	100	0/24/48	Abilities of daily life activity
	Veterans RAND 36-item health survey (VR-36)—Mental component score	100		Psychological Health
	Functional independence measure—Total	126		Overall life quality
	Functional independence measure—Cognitive function	35		Social Functioning
	Functional independence measure—Motor function	91		Abilities of daily life activity
	Craig handicap assessment and reporting technique (CHART)—Social interaction	100		Social Functioning
	Craig handicap assessment and reporting technique (CHART)—Mobility	100		Abilities of daily life activity
	Craig handicap assessment and reporting technique (CHART)—Cognitive independence	100		Psychological Health
	Craig handicap assessment and reporting technique (CHART)—Occupation	100		Social Functioning
	Craig handicap assessment and reporting technique (CHART)—Physical independence	100		Abilities of daily life activity
	Craig handicap assessment and reporting technique (CHART)—Economic self-sufficiency	100		Social Functioning
Szanton 2011 [47]	The activity of daily life difficulties (ADL)	5	0/24	Abilities of daily life activity
	The instrumental activity of daily life difficulties (IADL)	6		Abilities of daily life activity
	Health-related quality of life (Euro-QOL)	100		Overall life quality
	EuroQoL- 5 Dimensions (EQ-5D)	6		Overall life quality
	Fall efficacy	65		Psychological Health

**Table 3:** The League Table of the interventions for overall life quality of the disabled.

Active Exercise	−0.01 (−0.58, 0.54)	−0.01 (−0.57, 0.50)	−0.03 (−0.59, 0.47)
	Multi-disciplinary Program	−0.00 (−0.17, 0.16)	−0.03 (−0.19, 0.13)
		Psychosocial Support Program	−0.02 (−0.08, 0.04)
			Usual Care
	Drug Treatment	0.51 (−0.91, 1.95)	0.40 (−0.43, 1.23)
		Passive Therapy	−0.11 (−1.26, 1.02)
			Placebo

**Table 4:** The ranking of measures and probabilities of the interventions for the overall life quality of the disabled.

Treatment	Rank 1	Rank 2	Rank 3	Rank 4
Active Exercise	0.46	0.06	0.06	0.42
Multi-disciplinary Program	0.28	0.29	0.21	0.22
Psychosocial Support Program	0.22	0.41	0.29	0.08
Usual Care	0.04	0.24	0.43	0.28
Drug Treatment	0.09	0.19	0.73	
Passive Therapy	0.57	0.24	0.19	
Placebo	0.34	0.57	0.09	

**Table 5:** The results of the node splitting analysis.

Name	Direct Effect	Indirect Effect	Overall	*p*-Value
MP, PS	−0.07 (−0.31, 0.20)	0.02 (−0.18, 0.20)	−0.00 (−0.17, 0.16)	0.62
MP, UC	−0.01 (−0.19, 0.16)	−0.11 (−0.35, 0.16)	−0.03 (−0.19, 0.13)	0.55
PS, UC	−0.02 (−0.08, 0.04)	0.07 (−0.28, 0.37)	−0.02 (−0.08, 0.04)	0.62

MP: Multi-disciplinary Program; PS: Psychosocial Support Program; UC: Usual Care.

**Table 6:** The league table of the interventions for abilities of daily life activity of the disabled.

Active Exercise	−0.00 (−0.10, 0.08)	−0.00 (−0.10, 0.08)	−0.00 (−0.10, 0.08)	−0.00 (−0.10, 0.08)
	Multi-disciplinary Program	0.06 (−0.04, 0.17)	−0.01 (−0.04, 0.01)	−0.00 (−0.02, 0.02)
		Psychological Education	−0.08 (−0.19, 0.03)	−0.06 (−0.17, 0.04)
			Psychosocial Support Program	0.01 (−0.01, 0.04)
				Usual Care
		Drug Treatment	0.04 (−0.06, 0.14)	0.00 (−0.03, 0.03)
			Passive Therapy	−0.04 (−0.14, 0.06)
				Placebo

**Table 7:** The ranking of measures and probabilities of the interventions for abilities of daily life activity of the disabled.

Treatment	Rank 1	Rank 2	Rank 3	Rank 4	Rank 5
Active Exercise	0.04	0.1	0.03	0.08	0.75
Multi-disciplinary Program	0.04	0.47	0.35	0.12	0.01
Psychological Education	0.84	0.04	0.04	0.06	0.02
Psychosocial Support Program	0.01	0.04	0.11	0.63	0.21
Usual Care	0.07	0.35	0.47	0.11	0.01
Drug Treatment	0.14	0.41	0.45		
Passive Therapy	0.71	0.07	0.22		
Placebo	0.14	0.53	0.33		

**Table 8:** The results of the node splitting analysis.

Name	Direct Effect	Indirect Effect	Overall	*p*-Value
AE, MP	0.03 (−0.16, 0.19)	0.06 (−0.05, 0.18)	0.05 (−0.05, 0.15)	0.69
AE, UC	0.06 (−0.05, 0.19)	0.02 (−0.13, 0.20)	0.05 (−0.06, 0.15)	0.68
MP, PS	−0.19 (−0.53, 0.11)	−0.01 (−0.04, 0.02)	−0.01 (−0.04, 0.01)	0.28
MP, UC	−0.00 (−0.01, 0.02)	−0.03 (−0.17, 0.13)	−0.00 (−0.02, 0.02)	0.75
PS, UC	0.01 (−0.01, 0.03)	0.19 (−0.12, 0.48)	0.01 (−0.01, 0.04)	0.26

AE: Active Exercise; MP: Multi-disciplinary Program; PS: Psychosocial Support Program.

**Table 9:** The league table of the interventions for the psychological health of the disabled.

Active Exercise	0.00 (−0.40, 0.42)	0.05 (−0.57, 0.67)	0.12 (−0.35, 0.60)	−0.00 (−0.44, 0.44)
	Multi-disciplinary Program	0.05 (−0.42, 0.51)	0.12 (−0.19, 0.42)	−0.00 (−0.26, 0.25)
		Psychological Education	0.07 (−0.47, 0.63)	−0.05 (−0.57, 0.48)
			Psychosocial Support Program	−0.12 (−0.32, 0.08)
				Usual Care
		Drug Treatment	0.02 (−0.17, 0.21)	−0.01 (−0.07, 0.04)
			Passive Therapy	−0.04 (−0.22, 0.15)
				Placebo

**Table 10:** The ranking of measures and probabilities of the interventions for the psychological health of the disabled.

Treatment	Rank 1	Rank 2	Rank 3	Rank 4	Rank 5
Active Exercise	0.20	0.16	0.15	0.16	0.32
Multi-disciplinary Program	0.05	0.19	0.29	0.32	0.16
Psychological Education	0.32	0.16	0.12	0.13	0.27
Psychosocial Support Program	0.41	0.30	0.17	0.09	0.04
Usual Care	0.02	0.19	0.27	0.30	0.22
Drug Treatment	0.14	0.41	0.45		
Passive Therapy	0.71	0.07	0.22		
Placebo	0.14	0.53	0.33		

**Table 11:** The results of the node splitting analysis.

Name	Direct Effect	Indirect Effect	Overall	*p*-Value
AE, MP	−0.02 (−0.50, 0.47)	0.05 (−0.68, 0.83)	0.00 (−0.40, 0.42)	0.86
AE, UC	0.04 (−0.66, 0.74)	−0.03 (−0.60, 0.53)	−0.00 (−0.44, 0.44)	0.88
MP, PS	0.12 (−0.59, 0.82)	0.11 (−0.25, 0.47)	0.12 (−0.19, 0.42)	0.98
MP, UC	−0.01 (−0.31, 0.28)	0.02 (−0.53, 0.60)	−0.00 (−0.26, 0.25)	0.91
PS, UC	−0.12 (−0.34, 0.10)	−0.11 (−0.88, 0.66)	−0.12 (−0.32, 0.08)	0.98

AE: Active Exercise; MP: Multi-disciplinary Program; PS: Psychosocial Support Program; UC: Usual Care.

**Table 12:** The league table of the interventions for the social functioning of the disabled.

Active Exercise	0.02 (−1.14, 1.20)	0.05 (−1.29, 1.34)	0.28 (−0.61, 1.16)	0.01 (−0.83, 0.84)
	Multi-disciplinary Program	0.03 (−0.56, 0.58)	0.26 (−0.58, 1.10)	−0.01 (−0.81, 0.79)
		Psychological Education	0.23 (−0.80, 1.29)	−0.04 (−1.03, 0.99)
			Psychosocial Support Program	−0.27 (−0.52, −0.01)
				Usual Care

The bold means the result is statistically significant.

**Table 13:** The ranking of measures and probabilities of the interventions for the social functioning of the disabled.

Treatment	Rank 1	Rank 2	Rank 3	Rank 4	Rank 5
Active Exercise	0.19	0.16	0.16	0.14	0.35
Multi-disciplinary Program	0.10	0.21	0.21	0.29	0.19
Psychological Education	0.22	0.18	0.16	0.20	0.25
Psychosocial Support Program	0.49	0.27	0.18	0.06	0.00
Usual Care	0.00	0.19	0.29	0.31	0.20

## Data Availability

The data that support the findings of this study are available from the corresponding author, upon reasonable request.

## Supplementary Materials

The following are available online at https://www.mdpi.com/1660-4601/18/5/2406/s1, Supplementary file: The original data of PROMs.
